# Extracellular circulating miRNAs as stress-related signature to search and rescue dogs

**DOI:** 10.1038/s41598-022-07131-5

**Published:** 2022-02-25

**Authors:** Gabriella Guelfi, Martina Iaboni, Anna Sansone, Camilla Capaccia, Michele Matteo Santoro, Silvana Diverio

**Affiliations:** 1 Laboratory of Ethology and Animal Welfare (LEBA), Department of Veterinary Medicine, Università Degli Studi di Perugia, via San Costanzo 4, 0126 Perugia, Italy; 2 Department of Veterinary Medicine, Università Degli Studi di Perugia, via San Costanzo 4, 0126 Perugia, Italy; 3 Italian Military Corp of Guardia di Finanza, via Lungolago 46, 06061 Castiglione del Lago, PG Italy

**Keywords:** Biotechnology, Molecular biology, Physiology

## Abstract

Our research explores serum extracellular circulating miRNAs (ecmiRNAs) involved in dog stress response immediately after the search and rescue (SAR) of missing people. The experimental plan considers four arduous SAR simulations. The SAR dogs are trained by the Alpine School of the Military Force of Guardia di Finanza (Passo Rolle, Italy). The First SAR Trial analyzed dog serum samples at rest time (T0), and immediately after SAR performance (T1) using the miRNome-wide screening next-generation sequencing (NGS). T1 versus T0 NGS results revealed a different expression level of let-7a and let-7f. Subsequently, in a large sample size including: 1st (n = 6), 2nd (n = 6), 3rd (n = 6), and 4th (n = 4) trials, let-7a and let-7f were validated by qPCR. Bioinformatics analysis with TarBase (v.8) and the Diana-mirPath (v.3) revealed a functional role of let-7a and let-7f in the p53 pathway to restore cellular homeostasis. Let-7a and let-7f, highly expressed at T1, could stop MDMs-p53 inhibition inducing the p53 increase in level. In addition, let-7a and let-7f, via p53 post-transcriptional regulation, buffers p53 transcription spikes. During SAR stress, the possibility of p53 preconditioning could explain the phenomenon of “stress hardening” where the tolerance of particular stress increases after preconditioning.

## Introduction

Stress is essential for existence. Physiological stress responses are an adaptive system to cope with environmental changes, but a metabolic imbalance can be incurred if stress severity increases. The best strategies for animal stress counteracting rely on early diagnosis of stress-induced harms^1^.

Recent research suggests that stress is implicated in sensory biology^2,3^. Olfactory detection is a process exhibiting plasticity^4^, and a part of this plasticity depends on exposure to environmental stress conditions. Stressful experiences lead to alterations in dog olfaction affecting their performance when scent-based work is required.

Dogs are macrosmatic animals recruited for smell-based work in detecting cancer cells^5^, viral infections^6^, explosives^7^, drugs^8^, or missing people^9^. Search and rescue dogs are valuable after natural disasters, mass-casualty incidents, and for locating missing people. SAR dogs explore a large open-space, particularly shaped terrain in chaos and various weather conditions. Though SAR dogs must have an excellent physical status and are trained to work with an apt mental focus, SAR actions cause extreme physical and mental exhaustion^10,11^ in the dog. When the dog searches, he focuses on the smell of missing people, and while mild stress helps him focus on the goal, prolonged or too intense stress could negatively affect his performance or compromise his health. Bearing in mind that detection dog applications continue to grow, knowledge of the molecular mechanism regulating SAR dog stress to improve dog training and protect dog well-being is urgently needed.

In recent years, advances in epigenetic research, concerning heritable changes in environment-related gene expression that do not represent variations in the DNA sequence, have identified a new class of molecules known as microRNAs (miRNAs). MiRNAs are small single-stranded non-coding RNAs of approximately 22 nucleotides that can regulate gene expression in the post-transcriptional stage through interaction with target mRNAs, leading to translational inhibition or gene silencing. MiRNAs seem to have a high impact on stress signaling to restore homeostasis in sudden environmental changes, de facto stress conditions, altering miRNA biogenesis, modifying the expression of miRNA targets, and buffering transcription peaks^12^. MiRNAs bind to their target mRNAs and down-regulate translation thereby inhibiting protein production. Each miRNA can potentially regulate several hundred mRNAs, and tens of miRNAs may regulate each targeted mRNA.

Extracellular miRNAs (ecmiRNAs) secreted in body fluids, including blood plasma, urine, saliva, and semen, are excellent biomarkers. EcmiRNAs may mediate paracrine and endocrine communications between tissues and modulate distal cell survival and differentiation^13^. EcmiRNAs are exported and imported from cells and travel in biofluids protected by extracellular vesicles (EV)^14^ or bound to proteins (Argonaute-2 or high-density lipoproteins). Exosomes and microvesicles^15^ are the main EVs that contribute to miRNA transfer to facilitate intercellular communication with function as protective packaging for the delivery of controlled concentrations of miRNAs and guide targeting towards their destination. Exosome-mediated miRNA delivery is the most endogenous transport generated under physiological or pathological conditions.

EcmiRNAs were correlated with differences in lifestyle activities, such as exercise and environmental stressors, suggesting that ecmiRNAs may serve as circulating signatures of metabolic homeostasis^16^. Of late, ecmiRNAs significantly impacted medical research generating new molecular diagnostics hopes as biomarkers for homeostatic imbalance and disease states due to their abundance, cell-type specificity, and stability in most solid and liquid clinical specimens^17^.

Our research aim was to examine the SAR dog ecmiRNAs abilities to regulate homeostatic response to SAR stress. We planned a high-stress SAR simulation that required many of the skills dogs utilize when searching for missing persons after a natural disaster. The working dogs enrolled in this study belong to the Italian Military Forces of the Guardia di Finanza (GdF) and possess skills acquired during high professional training courses. Becoming a SAR dog requires immense expertise, and not every dog is up to this task. The NGS strategy was used to sequence dog serum miRNome at rest time and after SAR simulation.

We believe few observations on the role of ecmiRNAs as regulators of working dog stress are found in the literature. Nonetheless, in view of this powerful molecular approach, we have pioneered the diagnostics of working dog stress through the quantitative detection of minimally invasive ecmiRNA biomarkers that could improve training in the working dog as well as dog well-being.

## Methods

Study approval was provided by the Research Ethics Committee of the University of Perugia (report n.2018-21 of 11/12/2018) according to Italian Ministry of Health legislation^18^. All methods were carried out following relevant guidelines and regulations and the study was carried out in compliance with the ARRIVE guidelines. Informed consent is not required as no human subjects were included in the study.

### Animal enrolment

The dogs enrolled in the study were tested physically and behaviorally^18^. The day before SAR trials, all dogs were tested for routine health control by analyzing some blood biochemical parameters. The experiment included the dogs refer to the respective table (SM 1. SAR dog characteristics). Dog-handler teams were all specialized in the avalanche, surface, and rubble search at least by specific and increasing challenging training courses at the GdF Dog Breeding and Training Centre^18^ (Castiglione del Lago, Perugia, Italy) and the SAR Alpine School (Passo Rolle, Trento, Italy). All SAR dogs lived and worked with their handlers all year round to strengthen the dog-handler relationship, one of the most essential requirements for the success of a rescue operation.

### SAR trial

The simulated SAR trial required dogs to find a target odor (a hidden operator and his breath, simulating a missing person) on a rubble field (30 × 35 m), within a maximum time of 15 min. Before starting SAR trials dog handlers ignored where the operator was hidden. During the search, both dog and the handler were allowed to enter the field area. Simulated SAR performance included some potential stress factors, such as the presence of people working on the rubble area, shouting and making loud noises with hammer and shovel, and sources of smoke to simulate a real post-earthquake scenery. The SAR trial is considered successful when the handler raises his arm to communicate that his dog has signaled the target odor. If the dog does not find the hidden operator within the assigned time, the SAR trial is considered a failure. To safeguard the welfare of the SAR dogs, during all trials, the authors observed if the dogs showed signs of stress or anxiety according to the codes described in our previous studies^19,20^. The results of these observations were only used for practical monitoring of the level of stress during the field search and interrupting it if necessary.

### Sample collections time and experimental flowchart

#### Time points

All dog measures were evaluated in two experimental time points: T0—basal value at rest, immediately before the SAR trial; T1—immediately after the SAR trial. The points T0 and T1 allowed a comparison before and after the SAR trial.

#### Experimental flowchart

The research plan was divided into two experimental blocks; NGS SAR Trial and QPCR SAR Cluster, and both experimental blocks included T0 and T1 sampling times. NGS SAR Trial comprehended the 1st SAR trial, while QPCR SAR Cluster comprehended four SAR trials: 1st, 2nd, 3rd, and 4th. The four trials were held in different sessions, with different GdF dog-handler teams but the same experimental. The four trials included 22 dogs distributed respectively: 6 in the 1st, 6 in the 2nd, 6 in the 3rd, and 4 in the 4th Trial (see SM 1. SAR dog characteristics).

**Figure 1:**
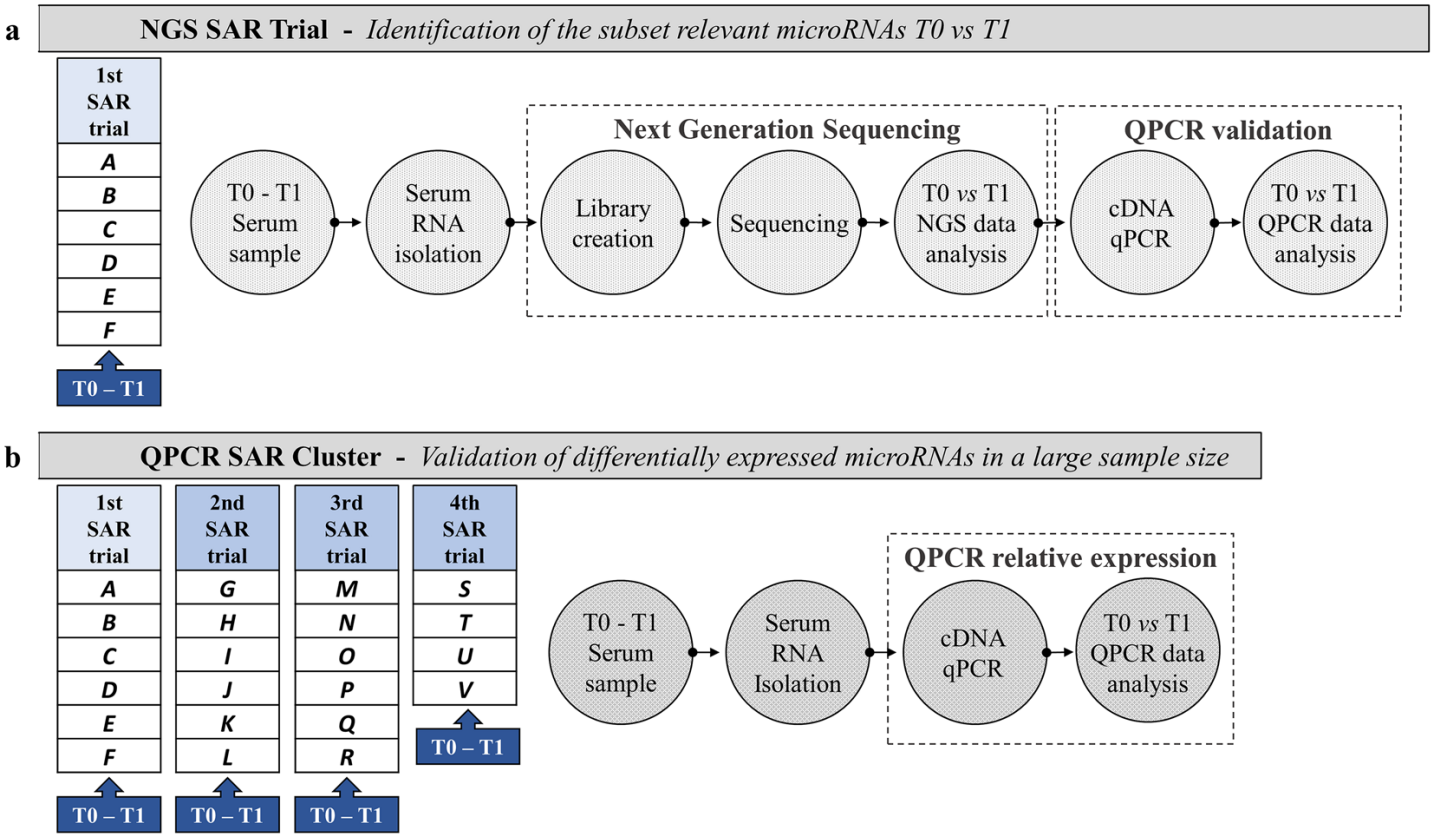
Research flowchart. The upper part of figure **(a)** shows the six dogs included in the NGS SAR Trial. The serum of six dogs was collected at two experimental time points (T0 and T1), RNA was extracted and processed for next-generation sequencing miRNome. The figure below **(b)** shows the second part of the research plan referred to as QPCR SAR Cluster. This part of the study includes blood dog sampling at T0 and T1 of the 2nd, 3rd, and 4th SAR trials. Serum RNA was extracted, reverse transcribed, and only the microRNAs differently expressed, detected in NGS SAR Trial, were validated by qPCR. The figure is created by G. Guelfi using PowerPoint 2021, Microsoft Corporation, USA.

NGS SAR Trial investigation was planned to evaluate dog serum miRNome and identify differently expressed ecmiRNAs between T0 and T1. QPCR SAR Cluster validated in large sample size, including the 1st, 2nd, 3rd, and 4th trials, ecmiRNAs with different expression profiles between T0 and T1 (according to the data obtained from NGS SAR Trial) (Fig. 1).

The day before the SAR, blood parameters were evaluated in all dogs included in the research. At experimental times, T0 and T1, heart rate, rectal temperature, serum cortisol level, and differential levels of serum microRNA expression were evaluated. The performance of the SAR dogs was examined throughout the SAR operation.

### Physiological parameter

The GdF veterinarian monitored heart rate (HR) in beats per minute with a stethoscope (3 M LITTMANN, Classic II SE, Milano, Italia) and measured rectal body temperature with a digital thermometer (Reckitt Benckiser SPA, MB Termo 7126500, Milano, Italia) at T0 and T1, in each SAR dog belonging to First SAR Trial, and QPCR SAR Cluster.

### Blood sampling and serum RNA purification

Blood sampling was taken following the routine health check protocol in the Gdf training program. During the GdF veterinarian procedures, the handler asked the dog to stand and stay still for 1 min while gently manipulating and distracting it. Next, the handler asked the dog to sit; simultaneously, the veterinarian collected the 3 mL of blood sample via the radial vein into Vacuette Z Serum Sep Clot Activator (GREINER BIO-ONE). After centrifugation (2000 × *g*, 10 min), serum was obtained and stored at − 80 °C until use. Hemolysis was controlled in all serum samples to prevent the release of microRNA contained in the blood cells altered ecmiRNA profile (SM 2. Hemolysis assessment during sample preparations). Each serum sample was separated into two collection tubes: one tube (300 µL) for analyzing biochemical parameters and one (200 µL) to explore differential miRNA expression profiles. Total RNA, including ecmiRNAs, was extracted from 200 µL of serum using the miRNeasy Serum/Plasma Kit (QIAGEN CLC bio, Aarhus, Denmark) according to the manufacturer’s instructions, with an elution volume of 14 µL (SM 3. RNA extraction and Spike-in for qPCR validations) and then stored at − 80 °C until use. MiRNA concentration was assessed using the Qubit Fluorometer 4 and the Qubit microRNA Assay Kit (THERMOFISHER SCIENTIFIC, Kandel, Germany).

### Spike-in to monitor RNA extraction, cDNA, and qPCR technical quality

**Figure 2:**
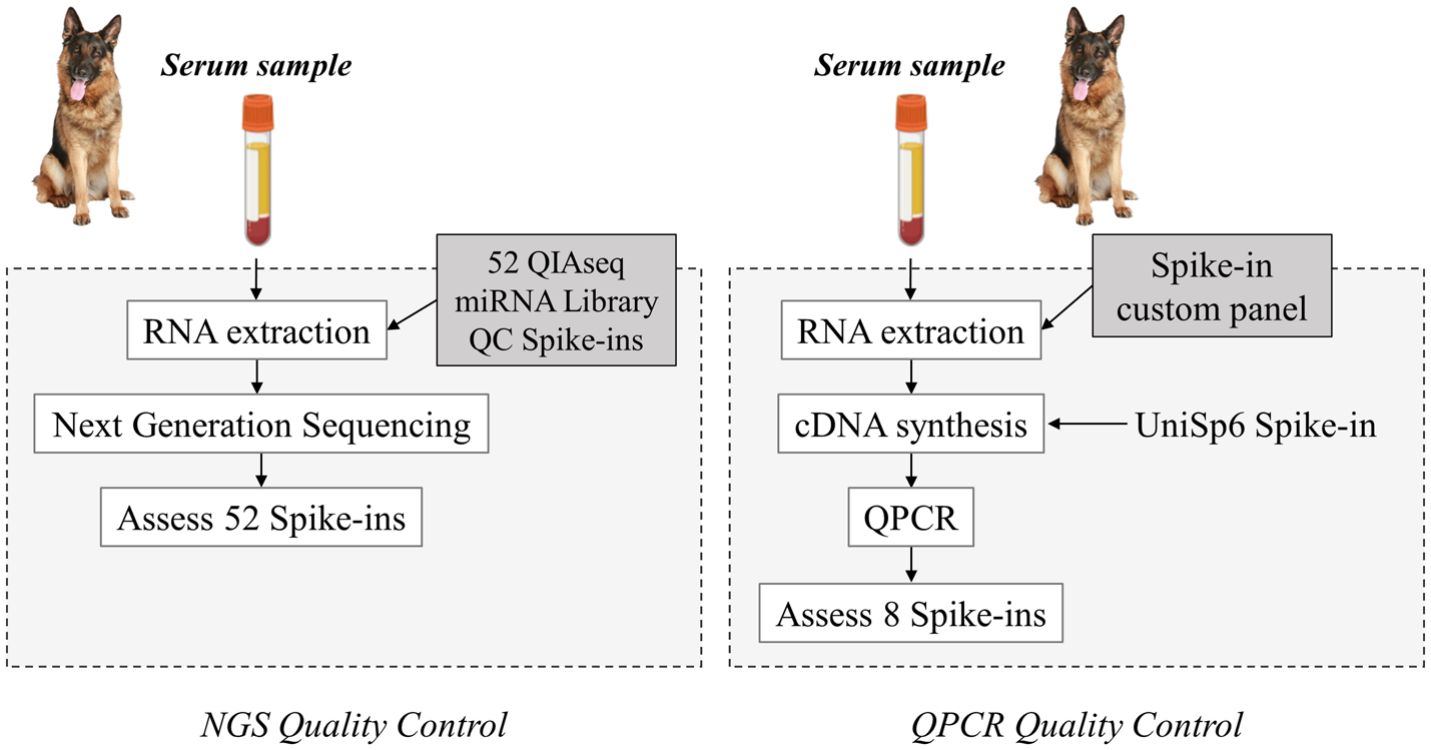
Quality control (QC) workflow. The NGS QC (left panel) was performed through a panel of 52 Spike-ins. The 52 RNA spike-in mix was added during RNA isolation to identify contamination and measure and validate assay parameters. In QPCR QC (right panel), 2 spike-in controls were added during RNA extraction to monitor the yield of RNA isolation, and 1 spike-in control was added to cDNA synthesis to monitor the efficiency of RT reactions. In addition, 3 spike-ins control PCR amplification. Spike-in synthetic oligonucleotides fragments were amplified and quantified by QPCR thanks to the respective primer pair. The figure is created by G. Guelfi using PowerPoint 2021, Microsoft Corporation, USA.

Spike-in was used to perform quality control (QC) of ecmiRNA from biofluid samples. The QC assessment is essential because it enables obtaining sensitive and reliable microRNA data from low RNA content samples. During sample preparation, the spike-in control oligonucleotide was added to observe the sample QC of the NGS SAR Trial and the QPCR SAR Cluster (Fig. 2). To assess the technical reproducibility and linearity of the mapped NGS reads, in the samples targeted for NGS, 1 µl of 52 QIAseq miRNA Library QC Spike-in (QIAGEN CLC bio, Aarhus, Denmark) was added during extraction, as suggested by QIAGEN NGS Services proprietary protocol. Whereas, in the QPCR SAR Cluster samples, the spike-ins UniSp2 and UniSp4 were added during the RNA extraction phase to assess the efficiency and yield of the RNA isolation as recommended by the manufacturer. UniSp6 spike-in was included in reverse-transcription reaction to monitor cDNA synthesis performance and check the presence of PCR inhibitors.

### MiRNA library preparation

EcmiRNA sequencing experiments and data analysis were conducted by QIAGEN Genomic Services (Hilden, Germany). Library preparation is the first step of next-generation sequencing. Once libraries were prepared, the following workflow step will be high-throughput sequencing. The RNA used in this step was extracted (as described in SM 1) by adding to the serum 1 μL of a 52 miRNA Library QC Spike-In mix (QIAGEN CLC bio, Aarhus, Denmark) (SM 4. NGS serum miRNA Spike-in). The library preparation was done using the QIAseq miRNA Library Kit. A total of 5 µl RNA was converted into miRNA NGS libraries. Adapters containing UMIs (Unique Molecular Identifiers) were ligated to the RNA. Then RNA was converted to cDNA. The cDNA was amplified using qPCR (22 cycles), and during the qPCR, indices were added. After qPCR, the samples were purified. Library preparation was quality controlled using capillary electrophoresis (Agilent DNA 1000 Chip). Based on the quality of the inserts and the concentration measurements, the libraries were pooled in equimolar ratios. The library pools were quantified using qPCR. The library pool was then sequenced on a NextSeq (Illumina) to obtain 19 M 1 × 75 bp reads. Raw data was de-multiplexed, and FASTQ files for each sample were generated using the bcl2fastq (Illumina).

### Next-generation sequencing

NGS Data analysis was performed via QIAGEN CLC Genomics Server v20.0.4 (Hilden, Germany). The miRNA-seq counts were normalized by a TMM (trimmed mean of M values) method^21^ to calculate the effective library sizes, which were then used as part of the per-sample normalization. Adapters containing Unique Molecular Identifiers (UMIs) were ligated to the RNA, and the RNA was then converted to cDNA. The 12 nt UMIs between the linker sequence and Illumina adapter sequence were extracted from each sample-demultiplexed sequencing reads, and the corresponding UMI annotated the reads. After removing all artificial sequences, we discarded reads with < 15 nt or > 55 nt. Reads with the same or 1 nt differences in their UMIs were grouped as UMI grouped reads, and reads from each UMI group were merged to produce a consensus sequence representing a single molecule from the starting RNA sample. These consensus reads were then aligned to miRBase v22 with a maximum mismatch of 2 nt, and the unmapped reads were subsequently mapped to the human genome GRCh38 (ENSEMBL).

### Quantitative validation of ecmiRNAs by RT-qPCR

RT-qPCR reactions were performed to validate the expression profiling of selected miRNAs following the NGS results filtered with max group means more than 10, FDR p-value less than 0.05, and standard p-value significance at < 0.05.

A first-strand cDNA synthesis reaction was conducted using miRCURY LNA RT Kit (QIAGEN CLC bio, Aarhus, Denmark). Purified RNA (10 ng) was reverse-transcribed, including artificial RNA Spike-in control UniSp6. RT reaction was carried out according to the manufacturer’s guidelines (SM 5. Reverse transcription reaction). QPCR amplifications were executed utilizing miRCURY LNA SYBR Green PCR Kits (QIAGEN CLC bio, Aarhus, Denmark), in a final volume of 20 μL according to the manufacturer’s recommendations (SM 6. QPCR ecmiRNA validations). QPCR reaction was performed with 3 μL cDNA (diluted 1:50), and 9 specific pair primers (QIAGEN CLC bio, Aarhus, Denmark): 3 kinds of spike-in control (SM 7. Spike-ins Quality control); 4 kinds of potentials endogenous control (EC) of serum samples (SM 8. Selection of potential EC miRNAs); and 2 target miRNAs. The 9 pair primers are listed in Table 1. The amplification was performed in the StepOnePlus real-Time PCR system (APPLIED BIOSYSTEMS, California, USA). Three technical replicates were performed for each biological sample, and the average Cq (Quantification cycle according to the MIQE guidelines^22^) the averaged values of triplicate Cq were calculated; no-template controls were included in the analysis check contamination. The StepOnePlus Real-Time PCR software plotted the fluorescence intensity against the number of cycles and provided the Cq value using the automatic method. The 2^-ΔCq method was used to calculate the relative expression of the target miRNAs^23^.

**Table 1:** List of primers used in qPCR.

Primer	Recommended for:	QIAGEN ID
UniSp2	Spike-in (RNA isolation efficiency assessment)	YP00203950
UniSp4	Spike-in (RNA Isolation efficiency assessment)	YP00203953
UniSp6	Spike-in (RT and PCR inhibitors assessment)	YP00203954
cfa-miR-320	Serum EC (qPCR normalized quantities)	YP00206042
cfa-miR-148a	Serum EC (qPCR normalized quantities)	YP00205867
cfa-miR-24	Serum EC (qPCR normalized quantities)	YP02114589
cfa-miR-23a	Serum EC (qPCR normalized quantities)	YP00205956
cfa-let-7a	Target miRNA validation	YP00205727
cfa-let-7f	Target miRNA validation	YP00204359

The resuspended primer mix contains a forward and reverse recognition sequence. The table reported Spike-in primers, serum EC, and differential target miRNA selected by NGS.

### Analysis of reference miRNA stability

MiRNA qPCR analysis requires data normalization with the best EC to minimize data variation that can mask or exaggerate biological changes. Among the analyzed EC miRNAs: miR-320, miR-148a, miR-24, miR-23a, the best ECs were estimated thanks to four algorithms: GeNorm, Normfinder, BestKeeper, and the comparative method ΔCt (Cq is the recent nomenclature of Ct); RefFinder integration tool was then used to compare and integrate the four algorithms results.

### Statistical analysis

RT-qPCR reactions were performed to validate the expression profiling of selected miRNAs following the NGS results filtered with max group means higher than 10, FDR p-value less than 0.05, and standard p-value significance at < 0.05. The quantification and statistical tests used were described in the figure legends and the methods section. No data were excluded from our studies. GraphPad Prism 8 (GraphPad Software Inc.) was used to plot all of the graphs and calculate statistical significance using a two-tailed Student’s t-test.

## Results

### Biochemical blood parameters and physiological data

All blood biochemical parameters are included within the canine reference range values (SM 9. Biochemical blood parameters). These results allow us to exclude a pathological state that limits the enrollment of some subjects in the study. The stress levels were monitored by measuring SAR dog cortisol blood level; all dogs showed at T1 higher cortisol levels than at T0 (140.33 ± 4.52 ng/mL and 80.61 ± 1.38 ng/mL, respectively). At T0, the mean heart rate (70–155 bpm) and rectal temperature (38.6–39.1 °C) were within the normality range.

### SAR dog performance evaluations

All trained dogs detected the presence of a missing person and his breath within the maximum time allowed (15 min). No significant differences were found in detection time between the trials.

### Serum RNA quality and quantity evaluation

The mean RNA integrity number (RIN) was 8.9 (range 8.5–9.0), the 260/280 ratio was 1.9 (range 1.88–2.00), and the 260/230 ratio was 2.1 (range 2.00–2.20) indicating the high purity of RNA preparation in every sample. Total RNA yield was not significantly different among samples. RNA concentrations ranged from 9 to 12 ng/µL. Minimal variations in total RNA content were corrected during reverse transcription using fixed RNA input.

### MiRNA-next generation sequencing data

NGS evaluated miRNA differential expression profiles at two different times: before SAR stress (T0) and after SAR stress (T1); the authors declare that all relevant data supporting the NGS findings are provided as a supplemental file (see the supplemental file—NGS data analysis). Differential expression analysis of ecmiRNAs revealed that only cfa-let-7f and cfa-let-7a have significantly upregulated expression levels at T1 versus T0 (Table 2).

**Table 2:** EcmiRNAs NGS data.

Name	T1 vs T0						
	Max group means*	Fold change	Log_2_ fold change	P-value	FDR** p-value	− log_10_ FDR p-value	Bonferroni
cfa-let-7f	3625.500	1.509	0.593	0.000002	0.000493	3.306839	0.000493
cfa-let-7a	7115.167	1.367	0.451	0.000253	0.032198	1.492165	0.054397

Data were filtered with max group means higher than 10, an FDR-p-value less than 0.05, and a standard p-value (significance at < 0.05). Differential expression analysis of serum miRNome at T1 vs T0 revealed that cfa-let-7f (FDR p-value < 0.001 applying Bonferroni correction for multiple tests 0.000493) and cfa-let-7 (FDR p-value < 0.05 applying Bonferroni correction for multiple tests 0.054397) were significantly upregulated.

*Max group means is the maximum of the average RPKM's (Reads Per Kilobase of transcript, per Million mapped reads).

**False discovery rate corrected p-value is the expected proportion of false-positive genes in a set of genes.

**Figure 3:**
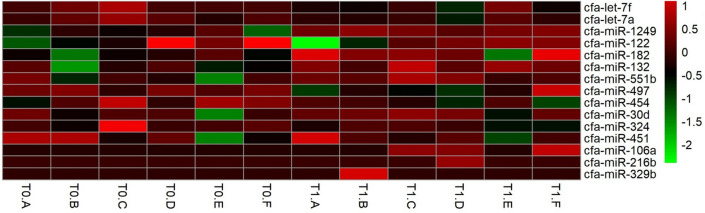
Volcano plot. The figure shows the relationship between the magnitude of the difference in expression values (fold change) and statistical p-values. The lower difference in expression, the nearer the point will be to the x-axis. The more significant the difference, the smaller the p-value, and the higher -log_10_ p-value. Thus, points for features with highly significant differences will lie high in the plot. The cfa-let-7f (0.000002 p-value) and cfa-let-7a in the plot (red) represent the differentially expressed ecmiRNAs with statistical significance (0.000253 p-value).

**Figure 4:**
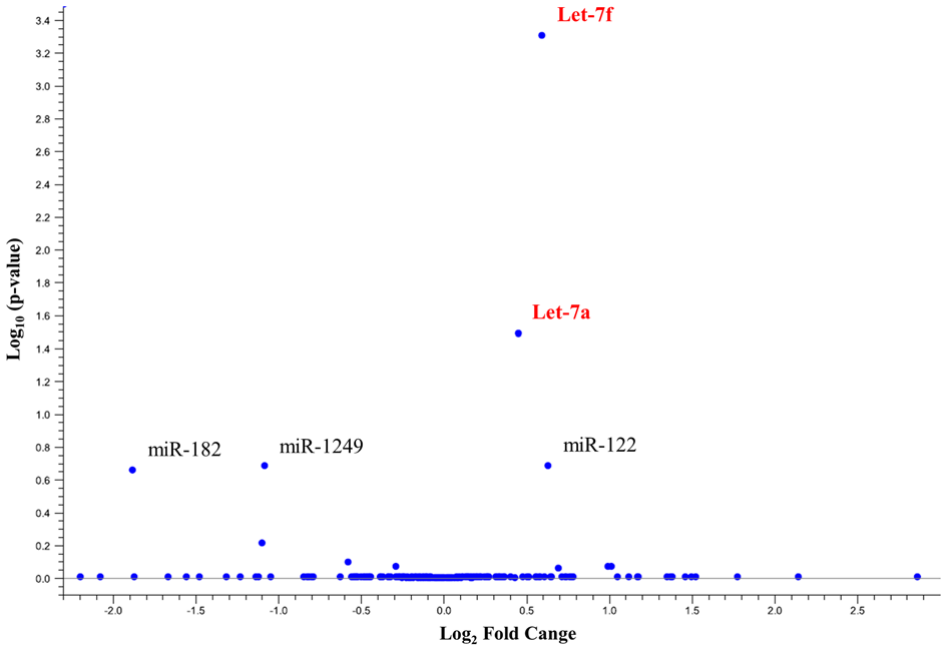
Heat map. Fifteen miRNAs with the highest FDR p-value across samples (T1 versus T0) were selected. Each row represents one miRNA, and each column represents one sample. The color represents the difference of the count value to the row mean. They are also called centered values. Red indicates high relative expression, and green indicates low relative expression.

The Volcano Plot (Fig. 3) was applied to identify differentially expressed ecmiRNAs with statistical significance. The heatmap (Fig. 4) showed the graphical representation of the fifteen miRNAs with the highest FDR p-value (T1 versus T0).

### Two-level control strategy of qPCR data

When the goal is to identify the expression level accurately, technical variability sources should be reduced. For this reason, we have adopted a two-level control strategy of qPCR data analysis: controlling the quality of the sample (hit 1) and identifying the best EC for data normalization (hit 2). By inspecting the amplification curves of UniSp 2, UniSp 4, UniSp 6, it was possible to identify and remove the outliers (Cq > 35), deriving from an incorrect technical procedure. Only two samples showed higher UniSp2 (Cq = 38), suggesting a problem in one of the RNA isolation procedure steps but repeating the isolation, the two samples yielded higher-purity RNA (hit 1).

**Figure 5:**
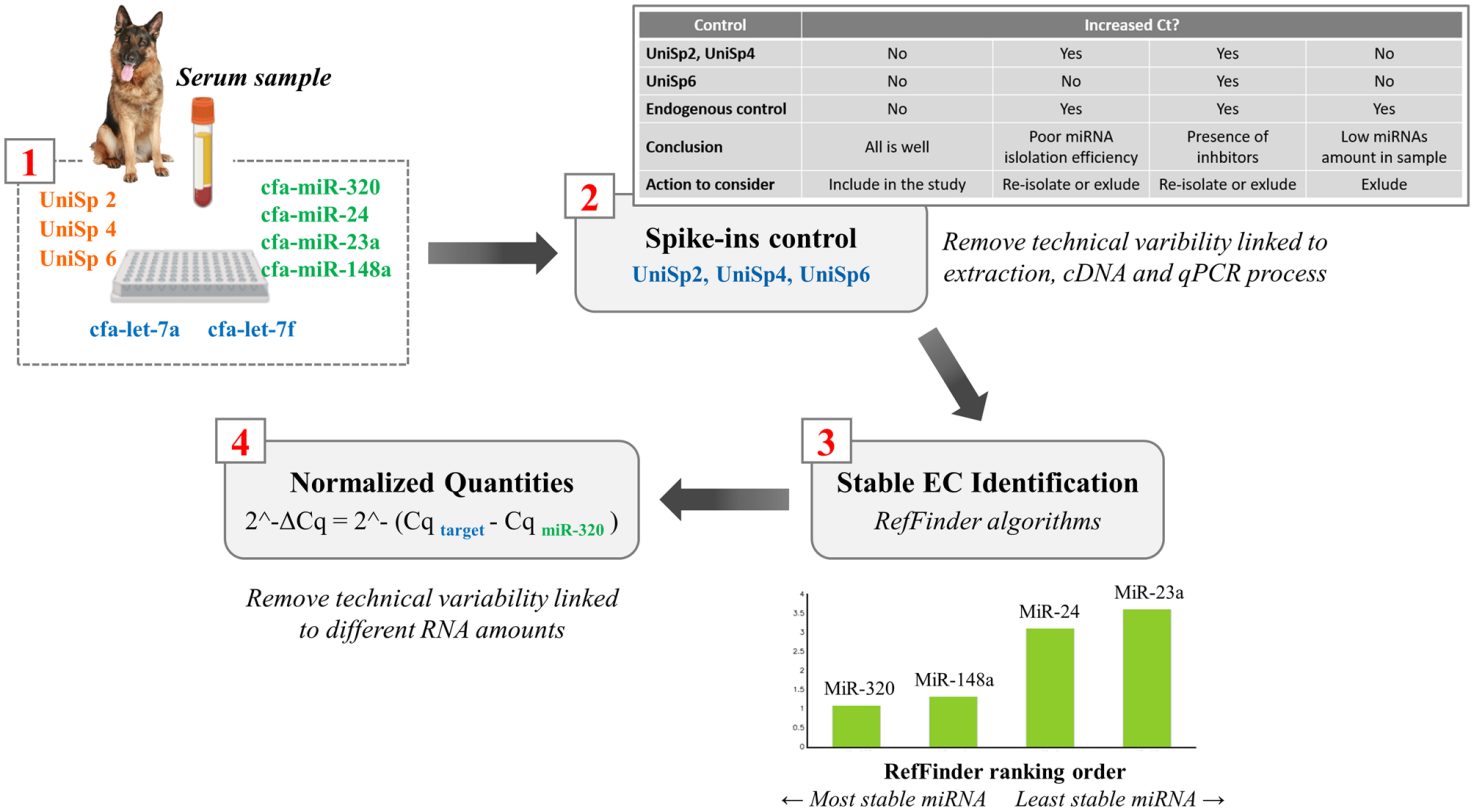
Control of qPCR data. In all serum sample, qPCR reaction was performed by amplifying 9 targets: let-7a and let-7f differentially expressed miRNAs, UniSp 2, 4, 6 Spike-in, and the EC miR-191, miR-423a, miR-93, and miR-425. UniSp2, UniSp4, UniSp6 enable confident interpretation, first data control (1 red). Spike-ins were compared, and the outlier was detected for sample exclusion or re-isolation. UniSp Cq was interpreted as described by QIAGEN in “Guidelines for Profiling Biofluid miRNA” (summarized in the table). The second data control was miRNAs normalization with the most stable miRNA EC (identified as mir-320). The figure is created by G. Guelfi using PowerPoint 2021, Microsoft Corporation, USA.

According to MIQE guidelines, we normalized qPCR data with the most stable EC miRNA to reduce experimental variability linked to the different quantities of the starting RNA. The best EC miRNA was identified using Delta Ct, BestKeeper, NormFinder, and GeNorm mathematical approaches. Subsequently, the data were analyzed using the four algorithms with RefFinder, which assigned a final ranking, identifying the best EC for qPCR data in the miR-320 (SM 10. Bioinformatics approaches for qPCR Endogenous miRNA selection). Target ecmiRNAs let-7a and let-7f Cq were normalized as follows: 2^ − ΔCq = 2^ − (Cq miRNA target – Cq miR-320) (hit 2) (Fig. 5).

### QPCR validation of let-7a and let-7f ecmiRNAs

**Figure 6:**
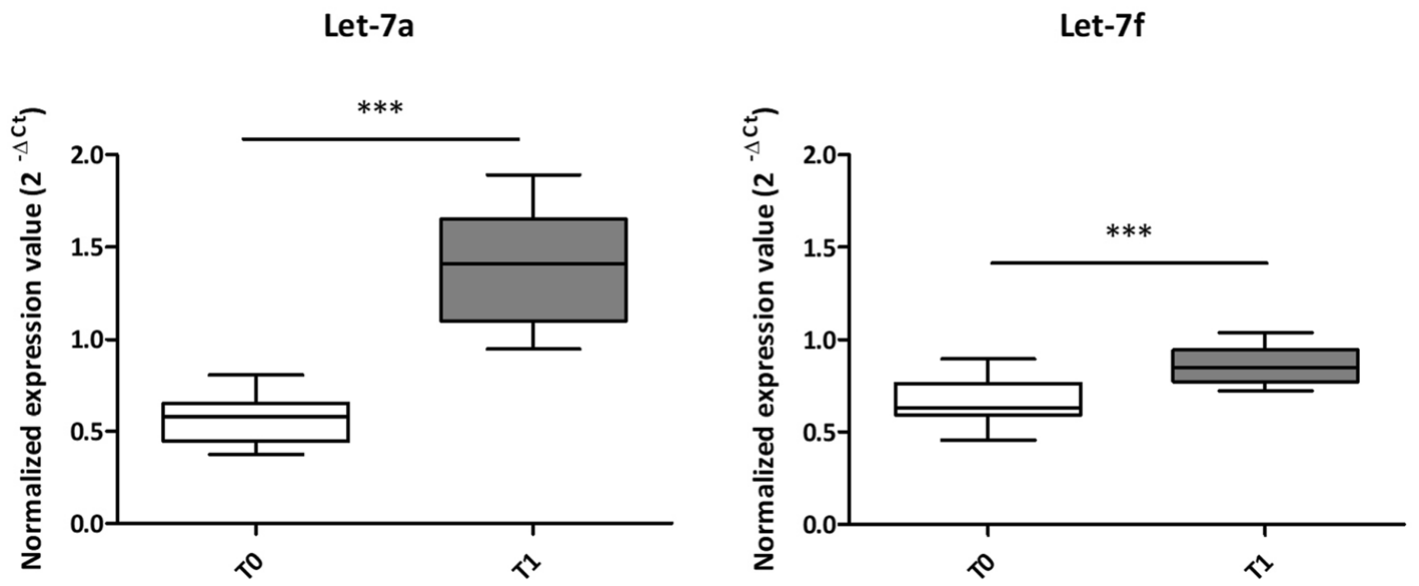
EcmiRNAs expression levels. The normalized expression values (y-axis) of the let-7a and let-7f ecmiRNAs were calculated with the 2^-ΔCq. The let-7a and let-7f graph show a white box (T0; 22 dog serum samples) and a grey box (T1; 22 dog serum samples). In the box plots, the thick central line represents the median; the top and bottom lines of the box represent the third quartile and the first quartile; whiskers indicate the variability in the data outside the upper and lower quartile. The figure shows the statistical significance of the overlap between the two groups determined using a two-tailed Student’s t-test; ***p ≤ 0.001.

The differential NGS miRNAs of First Trial dogs were chosen after removing the miRNAs that did not meet the NGS selection criteria (max group means higher than 10, FDR p-value less than 0.05, and standard p-value significance at < 0.05), and which had a low read count (≤ 10 reads) in paired samples. Then the p-value and FDR value of miRNA reads were calculated, and the ecmiRNAs with FDR < 0.05 were selected. At last, we obtained the relevant ecmiRNAs with onefold or more change. So, NGS revealed two ecmiRNAs: cfa-let-7a (FDR p-value < 0.05; applying Bonferroni correction 0.054397) and cfa-let-7f (FDR p-value < 0.001 applying Bonferroni correction 0.000493) significantly upregulated after SAR-related stress (T1) compared to the expression levels of rest time (T0). The NGS results of cfa-let-7a and cfa-let-7f were validated by qPCR in n = 22 including: First Trial (n = 6), 2nd (n = 6), 3rd (n = 6), and 4th (n = 4) Trial. The qPCR normalized expression values (2^ − ΔCq) of let-7a and let-7f ecmiRNAs showed a significant increase at T1 (immediately after SAR trial) compared to T0 (p ≤ 0.001) (Fig. 6).

### In silico microRNA target and pathway prediction

**Figure 7:**
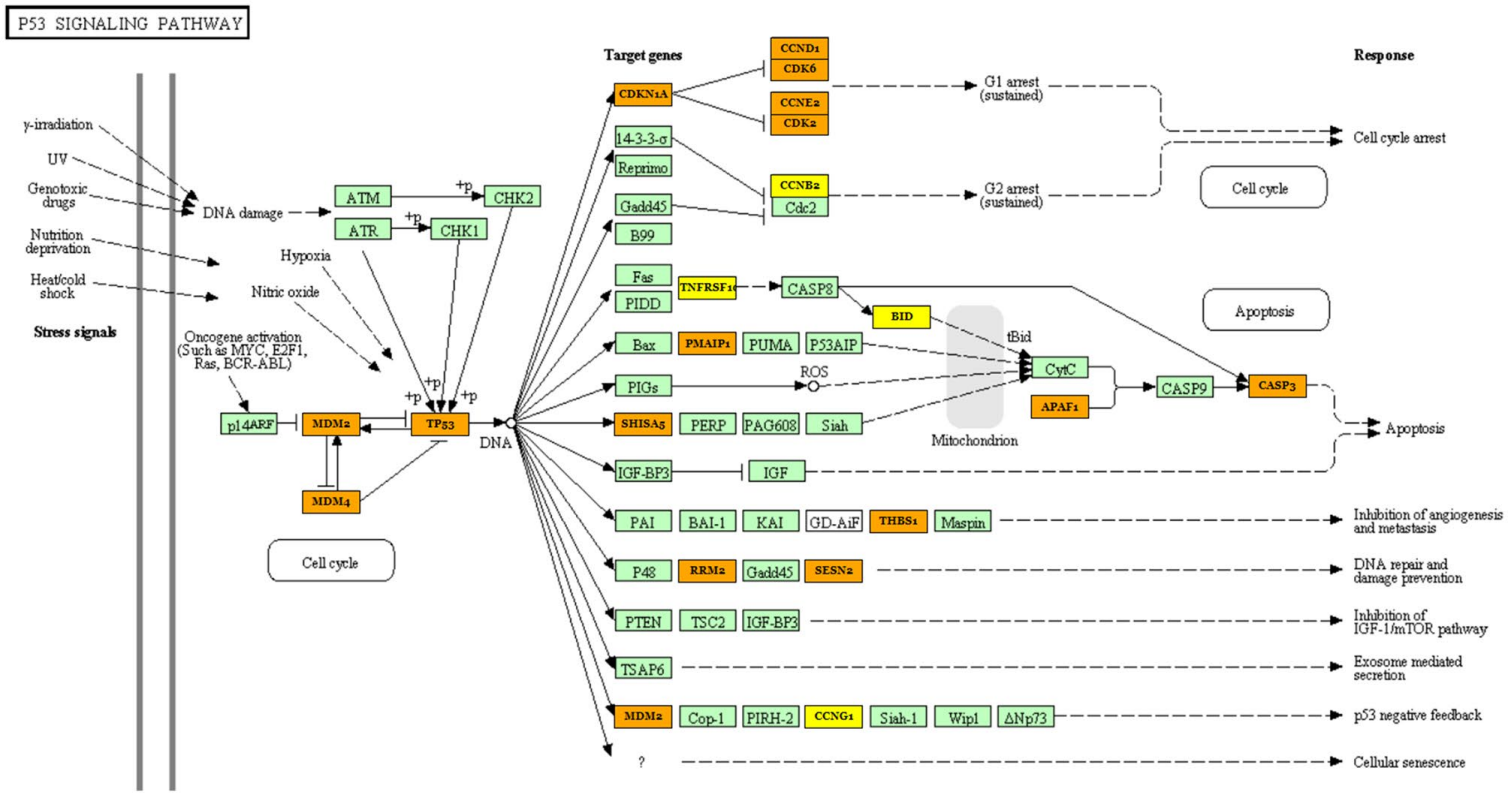
Pathway and targets prediction. The figure shows the associations between let-7a and let-7f ecmiRNAs and their putative p53 signaling pathway (p-value 0.0027432, and 23 gene interactions). The server offers two-gene labeling levels: yellow (gene targeted by 1 selected miRNA, let-7a), orange (gene targeted by 2 selected ecmiRNAs, let-7a and let-7f). Green color indicates genes that are not targeted by let-7a and let-7f Pathways analysis was performed using DIANA-miRPath v3.

MiRNA target prediction and functional analysis of let-7a and let-7f were performed with TarBase (v.8) and Diana-mirPath tool (v.3) implemented in the Kyoto Encyclopedia of Genes and Genomes (KEGG^24–26^), and StarBase v2.0^27^ revealing that these ecmiRNAs were involved in cell growth, proliferation, death, and cell survival (FDR ≤ 0.05, Fisher’s test). Our attention was focused on the p53 pathway (let-7a and let-7f ecmiRNAs union Diana-mirPath merge results showed p-value 0.0027432, and 23 gene interactions). The combinatorial effect of let-7a and let-7f in the p53 pathway is illustrated in Fig. 7. Let-7a and let-7f block the production of proteins transcribed by the target MDM2 and MDM4 and TP53 genes. The NMDs non-translation removes NMD-p53 inhibition restoring the p53 pathway. We like to underline that by merging in Diana-mirPath let-7a and let-7b, all the two ecmiRNAs have as mRNA targets: MDM2, MDM4, and TP53 (highlighted in orange in Fig. 7) (SM 11. Diana MiRPath and P53 KEGG pathway analysis).

## Discussion

Dogs are able to identify individuals by scent, although the exact molecular process is not fully understood. Dog variability in olfactory detection threshold is the product of genetic factors^28^ (olfactory receptor polymorphisms), physiological factors (hormone levels), life environment history (previous exposure to odorants, specific training^29^), and stress levels^30^. Recent research suggests that stress affects several aspects of olfactory detection^30^; odor perception especially is a process exhibiting plasticity^4^ altered by various endogenous signaling stress modulation. In light of this new evidence, our research hypothesizes that the SAR working dog may adapt the expression levels of ecmiRNAs as an epigenetic mechanism to balance SAR performance stress. SAR dogs are trained to find missing people, and their work can be time-consuming and complicated by endogenous and exogenous environmental stress factors. Dogs store these stressors inadvertently as they stay focused on their goal.

Based on dog serum next-generation sequencing, the first part of our study revealed let-7a and let-7f as ecmiRNAs capable of significantly differentiating dogs at rest and dogs immediately after SAR performance. In the second part of the study, involving a larger sample size of SAR dogs, we performed qPCR validation of let-7a and let-7f serum ecmiRNAs. The stringent filtering methods to improve the NGS data quality and the accurate qPCR data analysis allowed us to emphasize how let-7a and let-7f are involved in SAR stress response. Furthermore, we can underlie how from let-7a and let-7f ecmiRNA serum expression levels, it is possible to discriminate dogs that have performed SAR from those at rest. Concerning SAR stress, we focused our attention on ecmiRNAs expression profiling because, in humans, it was proven that in conditions of the stress response, cells restore their gene expression patterns modulating miRNA levels^31^. During unfavorable conditions, in an attempt to adapt to a reversible period of stress, a cell controls gene expression by post-transcriptional regulation^32^. Recent studies using genome-wide approaches and single-cell transcription measurements posit that in eukaryotic cells, miRNAs are ideal for buffering transcript surges during stress to restore gene expression programs via epigenetic regulation^33^. MiRNAs could ensure a steady gene expression level unless the stress signal is sustained long enough to increase the transcripts over a certain threshold^12^. For outlined reasons in recent years, there has been an enormous interest in investigating circulating miRNAs in blood plasma/serum as potential biomarkers for early diagnoses. Mori et al. (2019)^16^ suggests that ecmiRNAs may serve as circulating indicators of the physiological status and a tool for precision medicine targeted treatment.

Our research evaluated stress-mediated ecmiRNA levels as part of a natural physiological process, recovered in a short time, that occurs in SAR dogs. When SAR dogs spring into action, endogenous and exogenous stressors SAR-related temporarily affect canine physiological and behavioral status. Dog stress symptoms vary widely, including changes in the relationship with the handler, failure to perform work-related tasks, depression, and general signs of fear and anxiety^34^. Over the past decade, many open field tests have been conducted to identify the relationship between stress levels and the attitudinal and behavioral changes of the working dog. We are convinced that focusing on the molecular mechanism would help improve dog welfare, perform more efficient SAR, and probably identify a biomarker. This research is the first to investigate the implication of serum ecmiRNA in SAR performance stress modulation. However, a limitation of this study is not being able to provide detailed information on the quality and reproducibility assessment of NGS because, although accurately assessed, they are considered proprietary to the sequencing company. Our experimental study demonstrates that the physiological response to SAR stress through modulation of let-7a and let-7f levels contribute to the homeostatic recovery mechanism. Given the lack of knowledge on dog miRNA profiling and the high degree of evolutionary conservation of many miRNAs, in this scientific contribution, we disentangle the potential role of let-7a and let-7f in SAR stress based on scientific observations in humans^35^ and on in silico prediction of miRNA target and pathway. DIANA-miRPath v3.0 targets and pathway analysis implemented in the KEGG database showed that most of the let-7a and let-7f target genes were identified in the p53 stress signaling pathway. A crucial protein in the stress response is p53; the alteration of the TP53 gene or post-translational modification in the p53 protein can alter the response to cellular stress^36^. Canine p53 family members, like their human counterparts, are expressed at low levels under no stress conditions and rapidly upregulated under a stress condition^37^.

The p53-linked stress-sensing mechanism is activated by various intrinsic and extrinsic stress signals to promote cell cycle arrest and repair if stress damage is not severe^38^. Scientific research over the past decade suggests that the significant p53 stress response negative regulators are MDM2 and MDM4 homologs. In non-stress conditions, MDM2 and MDM4 inhibit p53 activity. Conversely, in a state of stress, when a p53 response is required to protect the cell, MDM2 and MDM4 stop p53 inhibition. In silico prediction of let-7a and let-7f targets revealed that these ecmiRNAs are involved in MDM2 and MDM4 post-transcriptional regulation. The upregulation of let-7a and let-7f, blocking MDM2 and MDM4, stops MDM-p53 inhibition inducing pro-stress signaling^39,40^. Let-7a and let-7f stop MDM-p53 inhibition and p53 increase in level depending on the degree of stress and preconditioning.

Post-translational modification of MDM2 to regulate the MDM2-p53 signaling axis to govern p53 activities in mammalian cellular stress was previously described by Malonia et al. (2015), which invites pharmaceutical control of the p53 pathway to protect against cellular stress^41^. On the other hand, it is proved that p53 can induce transcription of MDM2, generating a negative feedback loop^42^.

**Figure 8:**
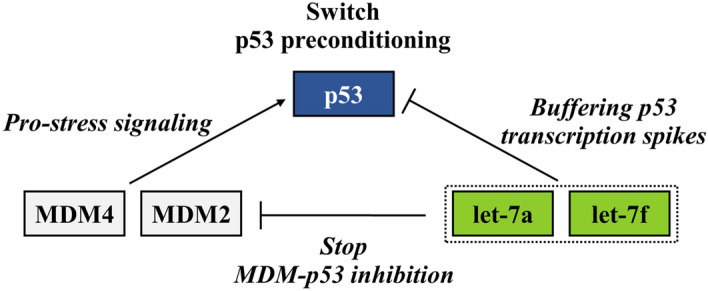
Tp53 preconditioning. The figure shows the hypothetical functional state of transient tolerance to SAR stress allowed by p53 preconditioning. The figure is created by G. Guelfi using PowerPoint 2021, Microsoft Corporation, USA.

In addition, let-7a and let-7f, via p53 post-transcriptional regulation, buffering p53 transcription spikes. Therefore, dog expertise could represent an environmental pretreatment that through preconditioning of p53, creates a cellular need-based gain that helps the dog during a stressful SAR (Fig. 8).

We suppose that SAR training developed the dog skills to exert a stress-protective epigenetic preconditioning mechanism by adapting the let-7a and let-7f miRNA levels. Epigenetic regulation is the basis of long-term changes, not written in the genome but directly associated with the environment. We assume the theory that let-7a and let-7f upregulation rapidly set the p53 preconditioning by triggering physiological stress reactions. The induction of miRNA expression upon stress might partly explain the phenomenon of “stress hardening”. Leung et al.^12^ and Kültz^43^ describe the physiological processes of miRNA upregulation under stress, to explain the phenomenon of “stress hardening” where the tolerance of same as previous increases after preconditioning. It should be emphasized that the long half-lives of ecmiRNAs, enable long-lasting gene regulation^12^. In the manuscript, we identify the SAR trial as a single stressor without distinguishing the importance of the components of the stressor. As Kültz argues, the cellular stress response is not stress-specific because a cell reacts to stress in response to the macromolecular damage sustained. Very few cellular responses directed at re-establishing homeostasis are stressor-specific^43^.

This research represents biomolecular advancement in understanding how working dogs adapt to SAR stress; it is not an in vitro functional study. Only reliable computational prediction information on the probable target genes and pathways involved in post-transcriptional let-7a let-7f regulation can be added. We preferred to evaluate single aspects, leaving further hypotheses to future works that will surely deepen comprehension of the functional role of let-7a and let-7f ecmiRNAs in physiological stress conditions concerning time recovery and performance complexity. In our opinion, it is fundamental to recognize the stressor outcome in working dogs to understand the behavioral consequence. It is critical to avoid that dogs^30^.

## Concluding remarks

NGS analysis and in silico prediction of SAR dog serum suggest that the let-7a and let-7f upregulation stop MDM-p53 inhibition, thus inducing p53 pro-stress signaling. The let-7a and let-7f upregulation could operate a double regulation, one inducing p53 pro-stress signaling activation and the other, in opposition, buffering p53 transcription spike. The balance of these two opposing molecular signals could mediate p53 preconditioning to the recovery of the homeostasis. We believe that SAR dogs, thanks to enriched environment experience of constant training, are able to control p53 preconditioning allowing behavior control and physiological stress recovery in arduous SAR performances. We hope this study will provide the cue to suggest interest in further research to improve dog well-being and dog SAR performance and, maybe explore the use of a circulating saliva miRNA as a non-invasive stress biomarker. This research is to be considered a first step in gaining further knowledge on how to build resilience in working dogs and better preserve their welfare.

## Supplementary Information

Supplementary Information 1.

Supplementary Information 2.
