# Supplementary material for: Extracellular circulating miRNAs as stress-related signature to search and rescue dogs
Source: Sci Rep. 2022 Feb 25;12:3213. doi: 10.1038/s41598-022-07131-5 (PMC8881509; doi:10.1038/s41598-022-07131-5)
Supplement: Supplementary file 1 — Supplementary Information 1. [file 41598_2022_7131_MOESM1_ESM.pdf]

Supplemental Material (SM)

SM 1. SAR dog characteristics.

The table shows the distribution and characteristics of the SAR dogs included in the trial. The dog identification (ID) is shown with a letter, and in parenthesis is indicated if male (M) or female (F).

1st Trial - NGS SAR Trial

Dog ID	A (F)	B (F)	C (F)	D (M)	(M)	F (M)
Age	3	2	2	2	3	3
Weight	25.4	22.7	26.1	27.3	30.3	18.3
Breed	Belgian Shepherd	Belgian Shepherd	Belgian Shepherd	Belgian Shepherd	Belgian Shepherd	Border Collie

2nd Trial - QPCR SAR Cluster

**Dog ID**	G (F)	H (F)	I (M)	j (M)	k (F)	L (M)
**Age**	2	2	3	3	3	3
**Weight**	21.4	25.4	28.3	27.6	15.4	27.2
**Breed**	Belgian Shepherd	Belgian Shepherd	Belgian Shepherd	Belgian Shepherd	Border Collie	Belgian Shepherd

3rd Trial - QPCR SAR Cluster

**Dog ID**	M (M)	N (F)	O (M)	P (F)	Q (M)	R (M)
**Age**	2	3	3	2	2	2
**Weight**	29.2	27.1	26.8	25.7	26.8	16.3
**Breed**	Belgian Shepherd	Belgian Shepherd	Belgian Shepherd	Belgian Shepherd	Belgian Shepherd	Border Collie

4th Trial - QPCR SAR Cluster

**Dog ID**	S (F)	T (M)	U (F)	V (F)
**Age**	2	3	3	3
**Weight**	27.1	17.2	27.6	28.1
**Breed**	Belgian Shepherd	Border Collie	Belgian Shepherd	Belgian Shepherd

SM 2. Hemolysis assessment during sample preparations

A significant source of variation in serum comes from contamination from cellular-derived ecmiRNA resulting from hemolysis. In serum samples, the level of hemolysis was measured using three methods. The first method (used in all processed serum samples) to assess hemolysis was a simple visual inspection of serum samples for pink discoloration, indicating free hemoglobin against a white background (carried out in the veterinarian field in all serum samples). The second method was used only for the samples processed with NGS (NGS SAR Trial samples) to monitor hemolysis and assessed the data from the red blood cell-specific miR-451a and the stable miR-23a determining the ratio miR-451a to miR-23a-3p (ΔCq; miR-23a-3p—miR-451a). The ΔCq levels lower than 5 in serum represents non-hemolyzed samples. If the ΔCq is close to or higher than 5, there is an increased risk of hemolysis. In all processed serum samples as a third method, recommended by QIAGEN for non-human serum samples, we evaluated hemoglobin concentration by the optical density at 414 nm (absorbance peak of free hemoglobin) using a NanoDrop 1000 spectrophotometer (THERMO SCIENTIFIC, Scoresby, Victoria, Australia). Samples were classified as hemolyzed if the oxyhemoglobin absorbance at 414 nm exceeded a value of 0.2 (third method). Serum samples (n = 22) hemolysis assessment, at the absorbance of 414 nm, showed a mean of 0.083, a standard deviation of 0.013, a minimum value of 0.068, and a maximum value of 0.12.

SM 3. RNA extraction and Spike-in for qPCR validations

Extraction was started by introducing 1 µL of spike-in mix*. The reagents and extraction method were provided by the MiRNeasy Serum/Plasma Extraction Kit (QIAGEN CLC bio, Aarhus, Denmark). At the end of extraction, RNA was eluted with 20 µl RNase-free water and centrifuged

for 1 min at maximum speed. RNA integrity was assessed using RNA 2000 and Small RNA Chips on a Bioanalyzer (AGILENT TECHNOLOGIES, CA, USA).

* Only for qPCR sample validations. UniSp2, UniSp4, UniSp5 are added only in samples validated for qPCR, while a mixture of 52 spike-in, QIAseq miRNA Library QC Spike-Ins solution was added to serum samples processed for NGS (procedure described in MS 5). Before starting the RNA isolation procedure for qPCR validations, the UniSp2, UniSp4, UniSp5 spike-in mixture was resuspended as described by the manufacturer. After qPCR, the Ct value obtained permitted the control for varying RNA purification yields. In qPCR, UniSp5 was not detected because it corresponds to weakly expressed microRNAs.

SM 4. NGS serum miRNA Spike-in

Before starting the RNA isolation procedure are added 1 μL of QIAseq miRNA Library QC Spike-Ins solution for 400-500 μL serum as suggested in QIAseq miRNA Library QC PCR QIAGEN handbook. 52 QIAseq miRNA Library QC Spike-ins mix was used to assess the technical reproducibility and linearity of the mapped NGS reads.

Spike-In name and sequence	Spike-In name and sequence
UniSp100 uugauucccaauccaagcaag	UniSp126 acaaacaccuuggauguucuu
UniSp101 uaccaaccuuucaucguuccc	UniSp127 aagcuuugcucguucauguuc
UniSp102 ucccaaauguagacaaagca	UniSp128 uaguccgguuuuggauacgug
UniSp103 ugaagcugccagcaugaucua	UniSp129 uuagaugaccaucaacaaacu
UniSp104 cagccaaggaugacuugccgg	UniSp130 ucuugcuuaaaugaguauucca
UniSp105 uccggcaaguugaccuuggcu	UniSp131 agcucugauaccaaaugauggaau
UniSp106 agaaucuugaugaugcugcau	UniSp132 ugaucucuucguacucuucuug
UniSp107 uuggcauucuguccaccucc	UniSp133 uguuuguuguacucggucuagu
UniSp108 uguguucucaggucaccccuu	UniSp134 uucuugcauauguucuuuauc
UniSp109 cgaaacuggugucgaccgaca	UniSp135 uccuguguuuccuuugaugcgugg
UniSp110 uucgaggccuauuaaaccucug	UniSp136 aucaguuucuuguucguuuca
UniSp111 uagaaugcuauuguaauccag	UniSp137 ucauggucagauccgucaucc
UniSp112 gguucguacguacacuguuca	UniSp138 ucgcucugauaccaaauugaug
UniSp113 uaaacuaaucacggaaaugca	UniSp139 uugaauugaagugcuugaauu
UniSp114 uuuuggaaauuuguccuuacg	UniSp140 ugacaugggacugccuaagcua
UniSp115 ugagccucugugguagcccuca	UniSp141 uaacuaaacauugguguagua
UniSp116 uuugcuuccagcuuuugucuc	UniSp142 uaagauccggacuacaacaaag
UniSp117 uugguuacccauauggccauc	UniSp143 uaauccuaccaauaacuucagc
UniSp118 uucgaugucuagcagugcca	UniSp144 gauggauaugucuucaaggac
UniSp119 ucuaagucuucuauugauguu	UniSp145 ccuuggagaaauaugcgucaa
UniSp120 uacgcauugaguuucguugcuu	UniSp146 uuaugucuuguugaucucaau
UniSp121 uggcuugguuuauguacaccg	UniSp147 uaaagucaauaauaccuugaag
UniSp122 uucugcuauguugcugcucau	UniSp148 uuuuuccucaaauuuauccaa
UniSp123 ugauuggaaauuucguugacu	UniSp149 augaauuuggaucuaauugag
UniSp124 ucuagcagcuguugagcaggu	UniSp150 auugguucaauucugguguug
UniSp125 uucuucgugaauaucuggcau	UniSp151 uaauuugguguuucuucgauc

SM 5. Reverse transcription reaction

The synthesis was performed using Mircury LNA MiRNA RT Assay (QIAGEN CLC bio, Aarhus, Denmark).

Component	Quantity (µL)
5x Mircury RT Reaction Buffer	2
RNase-free water	4.5
10x Mircury RT Enzyme Mix	1
Synthetic spike-ins UniSp6*	0.5
Template RNA (10 ng)	2
**Total reaction volume**	**10**

* UniSp6 RNA spike-in template was provided with the miRCURY LNA RT kit. UniSp6 was prepared as described by the kit manufacturer. The cDNA was incubated for 60 min at 42°C and 5 min at 95°C to inactivate the reverse transcriptase enzyme, then cool to 4°C. The cDNA samples were immediately placed on ice and then stored at -20 °C.

SM 6. QPCR ecmiRNA validations

For QPCR SAR Cluster samples, the qPCR reaction was performed with miRCURY LNA SYBR Green PCR Kits (QIAGEN CLC bio, Aarhus, Denmark). A hot-start procedure achieves high specificity and sensitivity in real-time PCR. This approach allows the room-temperature setup of the PCR reaction without the risk of primer–dimer formation.

Component	Quantity (µL)
2x miRCURY SYBR Green Master Mix	10
ROX Reference Dye	1
QPCR primer (listed in the manuscript in Tab. 1)	2
cDNA template (diluted 1:50)	3
RNase-free water	4
**Total reaction volume**	**20**

Mix the reaction thoroughly, dispense 10 µl from each well into qPCR plates, and spin the plate briefly. PCR cycling conditions included 15 min at 95 °C for enzyme PCR heat activation (at ambient temperature, the DNA Polymerase is kept inactive by antibody until the initial heat activation step); followed by 40 cycles of amplification: 15 sec 94 °C for denaturing double-stranded DNA, 15 sec 95 °C for annealing, and 15 sec 70 °C extension steps. Data acquisition should be performed during the annealing/extension step. Melting curve analysis 60-95 °C was performed to assess amplification specificity. The results interpreting spike-ins were performed referring to Qiagen suggestions. The PCR amplification efficiency was determined using the standard curve slope (efficiency = 10(−1/slope)−1). The slope of these graphs was utilized to determine the amplification efficiency. The PCR conditions were optimized to generate >95% PCR efficiency. Only reactions from 95 to 100% efficiency were included in the subsequent analysis.

SM 7. Spike-ins Quality control

The purpose of the RNA spike-in controls is to monitor the technical quality of RNA isolation and cDNA synthesis and to check PCR inhibitors in the sample.

Spike-in	Recommended for:	Introduced during:
UniSp2	RNA Isolation efficiency assessment	RNA extraction
UniSp4	RNA Isolation efficiency assessment	RNA extraction
UniSp6*	RT and PCR inhibitors assessment	cDNA

Custom panel of RNA sike-ins and relative primers (QIAGEN CLC bio, Aarhus, Denmark).

* Supplied with miRCURY LNA RT Kit

Spike-ins were amplified through PCR with relative forward and reverse primer mix. Following PCR, wells detecting the RNA spike-ins are compared, and outlier samples may be identified and considered for exclusion from further analysis. High variance >2 - 3 Cq difference within a dataset for a given spike-in reflects high variance in RNA yields or potential sporadic RNase contamination. Wells detecting spike-in UniSp6 were compared, and outlier samples (Unisp6 Cq > 30) were excluded from data analysis. UniSp2 is present at a concentration 100-fold higher than UniSp4. Therefore, UniSp2 should amplify at the level of very abundant miRNAs; UniSp4 should amplify approximately 6.6 cycles later than UniSp2.

SM 8. Selection of potential Endogenous Control ecmiRNAs

The ideal normalizer should be independent of biological variation, disease stage, or treatments, exhibiting similar storage stability, extraction properties, and quantification efficiency compared to the miRNA target. In dogs, there is a few conflicting information for ecmiRNA qPCR data normalization, so, following MIQE considerations, we provided a precise and detailed reference protocol for the EC selection and qPCR data normalization.

Based on the Coefficient of Variation (CV), we have identified the most stable ecmiRNAs in NGS output data (a low CV value indicates high stability). The eight most stable ecmiRNAs are listed below. The first four ecmiRNAs are selected as ECs of qPCR data normalization

NGS most stable ecmiRNAs

Name	Average CPM*	Standard Deviation	CV (%)
cfa-miR-320	20,856,591,832,380,200	276,304,603,143,253	13,2
cfa-miR-148a	15,129,704,676,218,600	2,299,015,091,529,200	15,1
cfa-miR-24	2,129,930,414,555,810	34,597,763,154,640,200	16,2
cfa-miR-23a	39,308,459,791,535,300	6,699,317,543,757,030	17,0
cfa-miR-148b	3,937,536,209,000,130	7,022,671,242,460,550	17,8
cfa-miR-27a	2,280,624,419,199,000	4,408,310,027,653,110	19,3
cfa-miR-27b	7,986,715,473,988,990	15,559,904,610,483,300	19,4
cfa-let-7b	7,763,614,745,823,620	15,571,372,136,108,100	20,0

* Counts per million

Selected ecmiRNAs for qPCR endogenous control

Name	MirBase accession
cfa-miR-320	MIMAT0006658
cfa-miR-148a	MIMAT0006622
cfa-miR-24	MIMAT0006614
cfa-miR-23a	MIMAT0006640

SM 9. Biochemical blood parameters

Blood biochemical parameters	Assessed values*	Normal values
Chlorine (Cl) mmol/l	105.35±3.2	105–115
Ferro (Fe) µmol/l	26.7±7.5	15–40
Phosphorus (P) mmol/l	1.37±0.12	0.84–2
Glutamic Oxaloacetic Transaminase (GOT) U/l	24.0± 3.0	23–44
Glutamic Pyruvic Transaminase (GPT) U/l	36.05±10.2	10–50
Alkaline Phosphatase (ALP) U/l	37.0±2.1	20–120

Glucose (mmol/l)	4.7±0.2	3.61–6.55
Lactate dehydrogenase (LDH)	174.7± 15.7	45–233
Non-Esterified Fatty Acids (NEFA) mmol/l	0.44± 0.03	0.4–0.7
Creatine Kinase (CK) U/l	99.25±12.6	30–120
Creatinine μmol/l	110.45±9.5	44.2–132.6

* Mean value±standard deviation

SM 10. Bioinformatics approaches for qPCR Endogenous Control ecmiRNA selection

The effect of a normalization process is highlighting true biological changes and eliminating, or at least reducing, the variability introduced during the experimental process linked to the different quantities of the starting RNA. According to MIQE guidelines, the correct way to normalize miRNA qPCR data is by using more stably expressed EC miRNAs. The most stable candidate EC miRNAs were identified using RefFinder, Delta Ct, BestKeeper, NormFinder, and GeNorm mathematical approaches. RefFinder integrates all the computational programs (geNorm, Normfinder, BestKeeper, and the comparative Delta-Ct method) to compare and rank the tested candidate reference genes. RefFinder, based on the rankings from each program, assigned an appropriate weight to an individual gene and calculated the geometric mean of their weights for the overall final ranking.

In all serum samples at T0 and T1, the Cq (Quantification cycle) of ECs were analyzed with software tools BestKeeper, NormFinder, geNorm, and Delta-Ct. The web-based tool RefFinder determined the final ranking order of ECs: miR-320 (geometric mean 1.19), miR-148a (geometric mean 1.41), miR-24 (geometric mean 3.22), and miR-23a (geometric mean 3.72).

	**RefFinder ranking order**			
**Method**	**1**	**2**	**3**	**4**
Bestkeeper	miR-148a	miR-320	miR-23a	miR-24
Normfinder	miR-148a	miR-320	miR-23a	miR-24
GeNorm	miR-320	miR-148a	miR-24	miR-23a
Delta Ct	miR-320	miR-148a	miR-24	miR-23a
**RefFinder Ranking order**	**miR-320**	**miR-148a**	**miR-24**	**miR-23a**

SM 11. Diana MiRPath and P53 KEGG pathway analysis

The table shows the two ecmiRNAs analyzed in Diana-mirPath. In the first column, miR-122 and miR-182 are joined with let-7a and let-7f. The second and third columns (left) indicate the analysis of the NGS data, the fourth and fifth columns report the p-value and gene interactions of two ecmiRNAs merged in the p53 pathway (Diana-miRPath).

miRNA Name	**NGS Data**		**P53 DIANA-miRPath**	
	FDR p-value	Bonferroni	P-value	Gene interaction
**let-7a**	**0.032198**	**0.054397**	**0.001956**	**19**
**let-7f**	**0.000493**	**0.000493**	**0.00635**	**19**
